# Common Pathology With Atypical Presentation: Acute Cholangitis

**DOI:** 10.7759/cureus.40747

**Published:** 2023-06-21

**Authors:** Aidan Farrell, Harshavardhan Sanekommu, Pranav N Shah

**Affiliations:** 1 Internal Medicine, Hackensack Meridian School of Medicine, Nutley, USA; 2 Internal Medicine, Jersey Shore University Medical Center, Neptune City, USA; 3 Radiology, Jersey Shore University Medical Center, Neptune City, USA

**Keywords:** biliary diseases, cholecystectomy, choledocholithiasis, endoscopy ercp, acute cholangitis

## Abstract

Acute cholangitis is a well-known biliary tree pathology most often encountered in patients with gallstone disease. When left untreated, acute cholangitis can lead to severe complications, including death. Therefore, identifying and properly treating acute cholangitis is crucial to avoiding such complications. This paper describes an 84-year-old female patient with acute cholangitis who presented with atypical symptoms of chest pain and cough. The patient was successfully treated with endoscopic retrograde cholangiopancreatography (ERCP), antibiotics, and ursodeoxycholic acid. We focus on this patient’s unique presentation to highlight the low incidence of Charcot’s triad and Reynold’s pentad in elderly patients and to emphasize the importance of formulating a broad differential in patients with non-specific symptoms.

## Introduction

Acute cholangitis is a disease of the biliary tree caused by acute infection and inflammation of the common bile duct [1]. Acute cholangitis is seen when biliary obstruction and biliary infection are present, with choledocholithiasis being the most common cause. There are less than 200,000 cases of acute cholangitis in America each year, with increased prevalence in populations prone to cholelithiasis [2]. Of patients in the hospital with gallbladder disease, 6-9% will be diagnosed with acute cholangitis [1]. The reported mortality rate of acute cholangitis is less than 10% after prompt treatment, with irreversible shock and multi-organ failure due to sepsis being the most common causes of death [2].

Acute cholangitis typically presents with a high fever, right upper quadrant abdominal pain, and jaundice (Charcot’s triad), with more severe cases also including hypotension and confusion (Reynold’s pentad) [1,2]. This paper will describe an atypical presentation of cough and right-sided chest pain in an elderly female diagnosed with acute cholangitis secondary to gallstones.

## Case presentation

This is an 84-year-old female with a past medical history of osteoporosis and hypothyroidism who presents with right-sided lower chest pain and a cough. The day before the presentation, the patient was sitting on her couch when she suddenly experienced an episode of dry cough that lasted for 20 minutes and eventually resolved on its own. The following night, the patient was sleeping when she woke up with right-sided chest pain and a cough. When she presented, she was afebrile, with a heart rate of 120 beats per minute, a respiratory rate of 25 breaths per minute, a blood pressure of 130/70 mmHg, and an oxygen saturation of 95% in room air.

On general appearance, the patient was diaphoretic and looked to be in acute distress. Her eyes were consistent with scleral icterus, and her skin was yellow in appearance. Inspection of the chest was within normal limits, and there was no focal tenderness or pain upon palpation. S1 and S2 were heard in all locations without an audible murmur or S3. She was tachycardic and in a regular rhythm. A lung examination revealed equal and bilateral breath sounds without any rales or crackles. Her abdomen was not distended, with no localized tenderness on both light and deep palpations. Significant labs included white blood cells (25.4/mm3) with 94.8% segmented neutrophils, 34% bandemia, total bilirubin 5.8 mg/dL, direct bilirubin 3.6 mg/dL, alkaline phosphatase (ALP) 144 U/L, aspartate aminotransferase (AST) 361 U/L, alanine transaminase (ALT) 201 U/L, lipase 33 U/L, and gamma-glutamyl transferase (GGT) 139 U/L. The chest x-ray was negative for acute cardiopulmonary pathology. No definitive infiltrates or lung consolidations were identified. An electrocardiogram (EKG) revealed sinus tachycardia and high-sensitivity troponin was negative three times.

**Figure 1:**
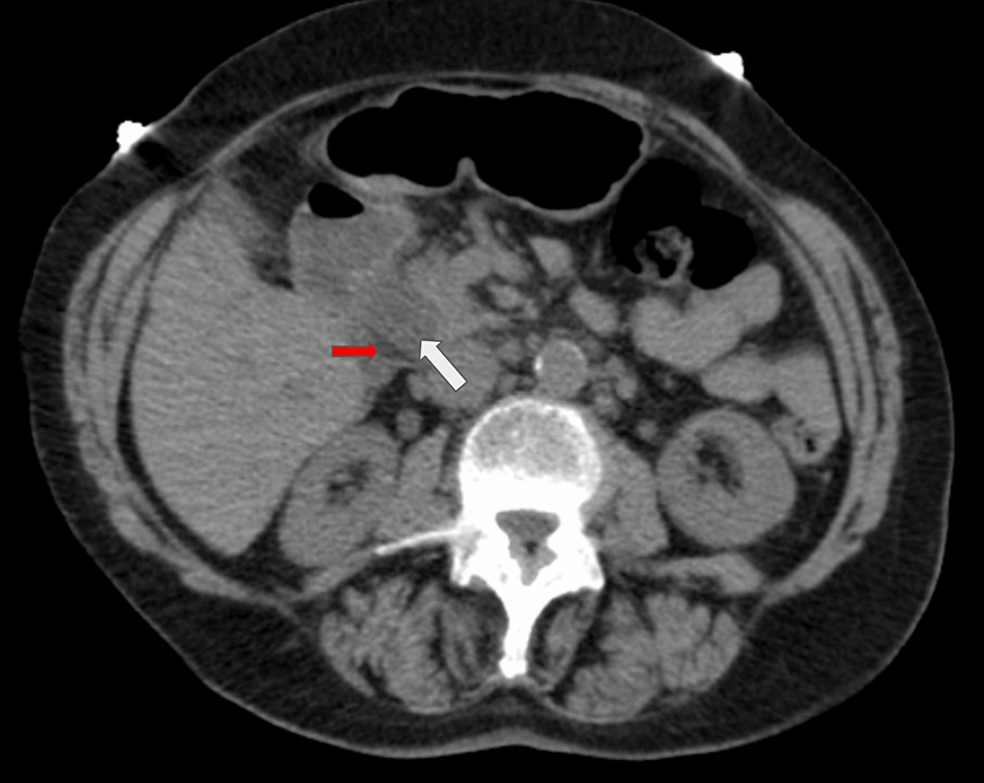
Axial CT of the abdomen without contrast shows intrapancreatic CBD dilation (28mm). The white arrow depicts a non-radiopaque calculus creating an incomplete obstruction of the bile duct. The red arrow depicts bile in the non-obstructed portion of the CBD. CBD: common bile duct

**Figure 2:**
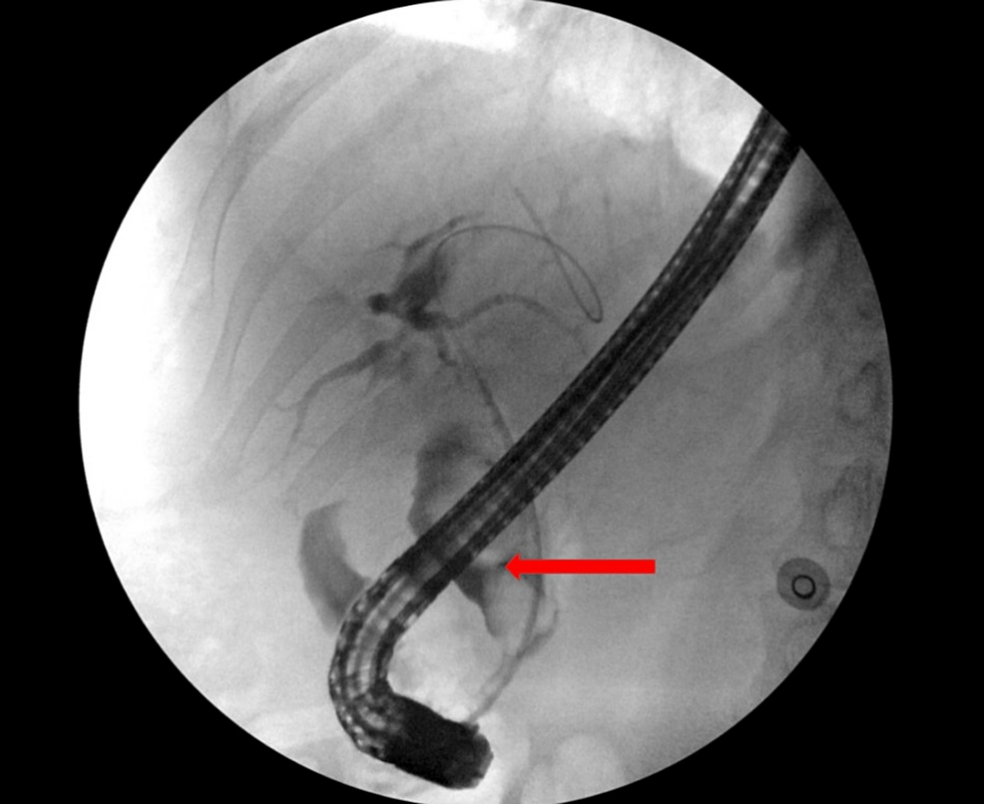
ERCP shows dilation and filling defects in the proximal and middle 1/3 of the common bile duct due to incomplete obstruction by two large calculi, with the largest measuring 20mm. ERCP: endoscopic retrograde cholangiopancreatography

The laboratory values were indicative of hepatobiliary pathology, and a computed tomography (CT) abdomen was done to further investigate. The imaging showed a marked dilation of the common bile duct of 2.9cm due to a large, non-radiopaque stone (Figure 1). She was diagnosed with acute cholangitis, and a sepsis protocol was initiated. She was started on broad-spectrum antibiotics (piperacillin, tazobactam, and vancomycin) and was given fluids. The next day, blood cultures resulted in bacteremia secondary to Klebsiella variicola. The patient underwent endoscopic retrograde cholangiopancreatography (ERCP), which showed two large stones in the lower one-third of the common bile duct (CBD), the largest measuring 20mm (Figure 2). One stone was successfully extracted with a stone retrieval basket, and a plastic biliary stent was placed. Her right-sided chest pain subsided during the hospital stay. Repeat blood cultures were negative, and the patient was discharged on oral amoxicillin and clavulanate. The patient was prescribed ursodeoxycholic acid and was scheduled for a repeat ERCP after 8 weeks. She underwent ERCP as an outpatient, with successful extraction of the stone. One month after the repeat ERCP, the patient’s jaundice had resolved, and the patient’s laboratory parameters were within normal limits.

## Discussion

Acute cholangitis carries a 10% mortality rate with timely diagnosis and appropriate treatment. The mortality rate increases to nearly 30% in severe cases that need emergent surgery [1]. Old age, high fever, leukocytosis, hyperbilirubinemia, hypoalbuminemia, and comorbidities such as malignancy, liver disease, and coagulopathies are all associated with a poor prognosis [2]. The complications of acute cholangitis are multi-organ failure and shock, leading to death due to sepsis [1]. After the acute stage of cholangitis, heart failure and pneumonia are also potential complications leading to mortality [1]. Hepatic abscess, liver failure, acute cholecystitis, portal vein thrombosis, acute pancreatitis, and acute liver failure are more commonly described complications of acute cholangitis [3].

The most common cause of acute cholangitis is biliary tract obstruction due to choledocholithiasis [1]. Though the formation of gallstones is multifactorial, well-studied risk factors include age greater than 40 years old, female sex, Native American ethnicity, high serum cholesterol levels, western diet, obesity, rapid weight loss (>1.5 kg/week), total parenteral nutrition (TPN), sedentary lifestyle, alcohol, and tobacco use, and diseases like diabetes, liver cirrhosis, and Crohn’s disease [4]. Besides gallstones, additional risk factors for acute cholangitis are neoplastic occlusion of the common bile duct, biliary stent obstruction, amyloid deposition, roundworm and tapeworm infection, and ERCP [2]. Considering her age and presentation, the cause of her acute pain and jaundice was thought to be a malignancy, alcohol use, or gallstones [5]. Our patient did not present with red flags like weight loss, night chills, abdominal pain, fever, or progressive jaundice. Her jaundice was more acute-onset, which made gallstones the suspected cause.

What made this case particularly challenging and unique was the patient's presentation. The Charcot triad (right upper quadrant pain, jaundice, and fever) has a sensitivity of 26.4% and a specificity of 95.9%. [3] Reynold’s pentad further involves Charcot’s triad, altered mental status, and hypotension, and it has a sensitivity of less than 5% [3]. In elderly patients, acute cholangitis is associated with vague symptoms and only 4.2% of patients present with the classic Charcot’s triad [6]. Our patient specifically presented to the hospital only complaining of acute right-sided chest pain and dry cough, likely due to inflammatory irritation of the diaphragm. Cough is a rare symptom of biliary tree pathology as it can irritate cough receptors in the diaphragm mediated by prostaglandin E, histamine, and bradykinin [7]. These intense bouts of cough probably led to pleurisy, which manifested as chest pain in our patient.

A definitive diagnosis of acute cholangitis requires visualization of purulent bile [3]. Because of this, tools like Charcot’s triad, Reynold’s pentad, and the Tokyo Guidelines are used as aids in diagnosis. Additionally, diagnostic imaging such as abdominal ultrasound, abdominal CT, and magnetic resonance cholangiopancreatography (MRCP) is helpful in determining the presence and location of biliary obstruction. Abdominal ultrasound is the imaging study of choice, with bile duct wall thickening, duct dilation, evidence of cholelithiasis, and pyogenic material all supporting a diagnosis of acute cholangitis. A CT scan is helpful in further visualizing inflammation and biliary duct dilation, but it also aids in assessing other causes of obstruction such as hepatic or pancreatic tumors, metastases, or abscesses [3]. When paired with the history and physical examination, a complete blood count (CBC), C-reactive protein (CRP), bilirubin, blood urea nitrogen (BUN), creatinine, and prothrombin time (PT) are laboratory tests that are helpful in diagnosing and assessing the severity of acute cholangitis [8]. Even though the presentation of our patient was vague, we were able to establish a diagnosis with the help of an abdominal CT and labs.

Acute cholangitis is treated with a variety of medical and/or surgical therapies. The Tokyo Guidelines were developed in 2006 to act as a framework for the proper management of cholangitis based on its severity. Acute cholangitis can be divided into three grades. Medical treatment may be sufficient for mild (grade one) acute cholangitis and usually begins with a third-generation cephalosporin (cefotaxime or ceftriaxone) or piperacillin/tazobactam [8]. Acute cholangitis that does not respond to medical therapy is described as moderate (grade two) [8]. Early surgical management with endoscopic or percutaneous drainage, followed by relieving the biliary obstruction via ERCP, is recommended in these patients [8]. Severe (grade three) acute cholangitis shows signs of end-organ failure and disseminated intravascular coagulation (DIC) [8]. Emergent surgical treatment is necessary for this subset of patients [8]. Stasis and the accumulation of bacteria in the biliary tract can cause bacteremia and sepsis when acute cholangitis is not treated. The Tokyo Guidelines place our patient in grade two acute cholangitis. Her elevated WBC count, radiographic evidence of biliary dilation and gallstones, total bilirubin greater than or equal to 5mg/dL, and age greater than or equal to 75 are the factors that contributed to her grading. Treatment for our patient included antibiotic therapy and early biliary drainage, which follows the Tokyo Guidelines recommendation for grade two acute cholangitis. More specifically, our patient’s course was complicated by bacteremia, which was managed with piperacillin/tazobactam and emergent ERCP. One of two gallstones was removed, and the patient was discharged with ursodiol and a second ERCP scheduled eight weeks later. Ursodiol was given to the patient due to its potential for gallstone dissolution and as conservative management until her follow-up ERCP was performed. Her second ERCP was successful.

## Conclusions

Gallstone disease is common in America, with acute cholangitis being a less common but deadly sequela of gallstones. Due to the numerous complications associated with untreated acute cholangitis, prompt recognition and intervention are crucial to reducing mortality. Elderly patients are more likely to have atypical presentations compared to those presenting with Charcot’s triad or Reynold’s pentad. Clinicians must be aware of this and should include biliary pathologies in the differential diagnosis of elderly patients with vague symptoms involving the abdomen, diaphragm, and thoracic cavity.
